# Management of first‐time patellar dislocation: The ESSKA 2024 formal consensus—Part 1

**DOI:** 10.1002/ksa.12620

**Published:** 2025-02-20

**Authors:** Lars Blønd, Marie Askenberger, Joanna Stephen, Ramazan Akmeşe, Peter Balcarek, Rene El Attal, Vasileios Chouliaras, Paolo Ferrua, Joan Minguell Monart, Geert Pagenstert, Petri Sillanpää, Manuel Vieira Da Silva, Jacek Walawski, Philippe Beaufils, Florian Dirisamer

**Affiliations:** ^1^ Orthopedic Department Zealand University Hospital Køge Denmark; ^2^ Aleris Hospital Copenhagen Denmark; ^3^ Section of Pediatric Orthopeadic Surgery Karolinska University Hospital in Solna Stockholm Sweden; ^4^ Hamlyn Centre Imperial College London London England; ^5^ Orthopaedics and Traumatology Department Çankaya Hospital Çankaya Ankara Turkey; ^6^ Department of Orthopedics and Traumatology Arcus Klinik Pforzheim Germany; ^7^ Orthopedics and Trauma Department Academic Teaching Hospital Feldkirch Austria; ^8^ Orthopaedic Department General Hospital of Arta Arta Greece; ^9^ Dipartimento di Scienze Biomediche per la Salute (SCIBIS) Università degli Studi di Milano Milan Italy; ^10^ I Clinica Ortopedica‐ASST Gaetano Pini CTO Milan Italy; ^11^ Orthopaedic Department Vall d'Hebron University Hospital Barcelona Spain; ^12^ CLARAHOF Clinic of Orthopaedic Surgery University of Basel Basel Switzerland; ^13^ Department of Orthopedics and Trauma Pihlajalinna Hospital Tampere Finland; ^14^ Orthopedic Department Trofa Saúde Hospital Braga Centro Braga Portugal; ^15^ Orthopedic Department ŻagielMed Hospital and MSWiA Hospital Lublin Poland; ^16^ Centre Médical, Fondation Norbert Metz 76 Luxembourg Luxembourg; ^17^ Orthopädie und Sportchirurgie Linz Puchenau Austria

**Keywords:** complications, first‐time patellar dislocation, formal, osteochondral lesion, patellar instability, rehabilitation

## Abstract

**Purpose:**

To provide recommendations for the treatment of patients with first‐time patellar dislocation (FTPD). Part I focused on clinical presentation, symptoms, diagnosis, evaluation and imaging.

**Methods:**

Fifty‐four orthopaedic surgeons and one physiotherapist from 20 countries across Europe were involved in the consensus, which was the FTPD. The consensus was performed according to the European Society for Sports Traumatology, Knee Surgery and Arthroscopy consensus methodology. The steering group designed the questions and prepared the statements based on the experience of the experts and the evidence in the literature. The statements were evaluated by the ratings of the peer‐review groups before a final consensus was released.

**Results:**

The consensus consists of 32 questions and statements, 13 of which will be reviewed in Part 1 of the review. There is an inverse correlation between the intensity of trauma leading to FTPD and the underlying pathoanatomic risk factors, meaning that low trauma intensity usually indicates more severe underlying abnormalities. In addition to the clinical investigation, patient age, family history, bilateral symptoms of instability and injury mechanism should be evaluated. However, reliance can be placed not only on clinical examination but also on magnetic resonance imaging scans as soon as possible, which are considered mandatory for evaluating predisposing factors such as trochlear dysplasia and patella alta and for detecting osteochondral lesions, with the exception of asymptomatic patients. Importantly, it must be recognized that in addition to recurrent instability, which affects approximately 25% of patients, a variety of symptoms are experienced by 50% of patients, such as pain, swelling, giving way, functional and psychological limitations, and a reduction in sports participation, all of which reduce their quality of life. The complications after medial patellofemoral ligament reconstruction in patients with FTPD have not yet been established; however, we know from cohorts of heterogeneous patients that the most common complications are patellofemoral pain, a reduced range of motion and patellar fracture. In total, there were 13 statements that were all accepted and achieved, 6 with strong agreements and 7 with relative agreements. The general median agreement was 8 (range 7–9). None were graded A, two were graded B, seven were graded C and 4 were graded D.

**Conclusion:**

In relation to the management of patients with first‐time patellar luxation, we have worked with 13 questions and based on these we have achieved consensus on 13 statements.

**Level of Evidence:**

Level I, consensus.

**Abbreviations**
AP: anterior posterior
BPII: Banff Patellofemoral Instability Instrument
CT: computed tomography
ESSKA: European Society for Sports Traumatology, Knee Surgery and Arthroscopy
FTPD: first‐time patellar dislocation
MPFL: medial patellofemoral ligament
MRI: magnetic resonance imaging
NPI: Norwich Patella Instablity outcome score

## INTRODUCTION

First‐time patellar dislocation (FTPD) as defined below is a common orthopaedic problem and this consensus handles the treatment covering all ages. In patients under 15 years of age, it is the most common cause of knee hemarthrosis. Patients with dislocated patellae are typically transferred acutely to the emergency room by ambulance, or they are seen in the office weeks after the injury for the first time, or at any point in between. This demonstrates the wide variety of this type of injury, ranging from dramatic and acute to less severe. The reason for this is the numerous different factors and circumstances that lead to one final condition—patellar dislocation. Therefore, the symptoms of patellar instability can have completely different histories and pathogeneses, which sometimes make this field complex.

In recent years, increasing knowledge has been gained in understanding the patellofemoral joint. Risk factors are continuously clarified and are relatively clearly defined [11, 21, 22]. Similarly, the biomechanics leading to patellar instability are delineated step by step, and new therapeutic strategies have been established. Our treatment, especially surgical treatment, has therefore changed substantially in recent years, and we are making good progress with respect to clinical data.

This consensus encompasses FTPD, and when we searched the literature, we identified some inconsistencies in the definition. This can potentially lead to incomparable data and scientific fraud; therefore, the consensus group suggests a simple definition to be used in future scientific projects.

“FTPD is the first‐time event when the patella completely leaves the trochlear groove. The event has to be confirmed clinically and/or radiologically.”

This unification will help to obtain comparable data and eventually clarify some data variations in the literature. The real incidence of FTPD, for example, is somewhat unclear, and the available data are heterogeneous due to differences in the inclusion criteria. The incidence of FTPD is age‐related and is highest during growth spurt. It usually occurs in patients younger than 18 years of age, with a tendency toward female patients. FTPDs in adults older than 25 years are uncommon. The reported overall incidence varies between 10 and 150/100,000 patient years and may be region‐specific, as the reported numbers have high deviations.

Despite a huge number of publications in the field of patellofemoral instability, relatively few studies with high scientific level exist, and many open questions remain. This is where a consensus project based upon a strict scientific methodology can provide valuable guidance and recommendations for general orthopaedic surgeons, orthopaedic specialists, trauma surgeons and also be relevant for physiotherapists. The purpose of this method is to combine scientific evidence and expert opinions in the field and to obtain this peer‐reviewed conclusion with the highest level of agreement. This can be useful as a guideline for everyone working in this field.

Given that there are region‐specific differences in health systems throughout Europe, a consensus cannot fully respect all these local aspects. However, owing to the strict methodology, this approach provides an ‘ideal’ model for a diagnostic or therapeutic approach and has the potential to be widely implemented throughout Europe.

FTPD is the starting point of every instance of patellar instability, and either it is a single event or it is followed by recurrent dislocations or subluxations with or without pain. Even ongoing symptoms such as patellofemoral pain or a limitation of knee‐related quality of life can be expected [14, 20]. The risk factors for FTPD have been substantially investigated and are well established, and they do not differ from those of chronic cases. There are anatomical risk factors and others such as age, hyperlaxity and the level of force causing the patella to dislocate (trauma intensity) [16] (see Table 1).

**Table 1:** Risk factors for FTPD.

Anatomical	Others
Trochlea dysplasia	Age
Patella alta	Hyperlaxity
Coronal and torsional (frontal) malalignment	Trauma intensity
Lateral position of tibial tubercle	

Abbreviation: FTPD, first‐time patellar dislocation.

Establishing and proposing an optimal individualized treatment for a patient is influenced by many factors. The patient's medical history (e.g., intensity of trauma leading to FTPD and bilateral instability), as well as one or more predisposal factors and the patient's demands must also be considered when a therapeutic strategy is being planned. Therefore, the aetiology of FTPD is considered extremely important, and decision‐making must be individualized and not based exclusively on clinical or radiological measurements.

Interestingly, there are more open questions about FTPD than about chronic cases. Despite the clear definition of FTPD, the clinical question of whether to treat this disease surgically remained unsolved. Essential questions and statements on this topic were developed by The ESSKA committee for patellar instability to combine the available literature and clinical experience.

Part I focused on clinical presentation, symptoms, diagnosis, evaluation, imaging and complications. Part II focused on nonoperative treatment, bracing, rehabilitation, indications and surgical strategies.

## MATERIALS AND METHODS

The European Society for Sports Traumatology, Knee Surgery and Arthroscopy (ESSKA) FTPD consensus process was initiated in April 2022. The method was based on the ‘formal consensus process’ as described by the French National Healthcare Institution (Haute Autorité de Santé [HAS]) and was specifically published by ESSKA [4, 5]. Briefly, a total of 55 European surgeons and scientists were involved in the process, comprising steering (14 experts), rating (24 experts) and peer review groups (17 orthopaedic surgeons). The two principal objectives of the Steering group were to (A) devise a framework of questions suitable for consensus and educational purposes and (B) thoroughly evaluate the scientific literature and combine it with expert opinion to produce robust statements. The steering group was initially subdivided into ‘questions’ and ‘literature’ groups. The two groups first worked independently to avoid bias, and after the questions were defined, all members of the steering group were involved in answering these questions based on the literature and expert experience.

### Question group

The question group was comprised of three experts who formulated a series of enquiries to cover the relevant and important aspects of FTPD. The questions were developed by those three after one of the members had prepared an agenda for discussion. Through online meetings and e‐mail correspondence, 32 questions were prepared after some were added and some were omitted. Since then, no new questions have been added, and none have been cancelled.

### Literature group

A literature group, comprising the remaining members of the steering group, conducted a literature search between November 2020 and May 2022, including PubMed, Google Scholar and EMBASE, according to keywords relevant to each specific question. The title and abstract of each reference were evaluated, and any relevant article was then obtained in full for the steering group to summarize as a brief report. Peer‐reviewed clinical studies with levels of evidence ranging from 1 to 5 were included in this analysis. Only papers published in English were considered.

### Number of questions‐statements

The question group designed a list of 32 questions presented for the rating group.

Part 1 focuses on clinical presentation, symptoms, diagnosis, evaluation, imaging and complications and questions 1–9 and 27–29 were included. Question 2 is separated into 2 questions and two statements with different grades. Part II focuses on nonoperative treatment, bracing, rehabilitation, indications and surgical strategies and contains questions 10 to 26.

Each question was designated based on its scientific level, that is, Grades A, B, C or D. A—high scientific level, B—scientific presumption, C—low scientific level and D—expert opinion.

### Rating group

The statements were rated by a group of 24 patellofemoral surgeons across European countries. Each expert was asked to evaluate all pairings by using a 1‐ to 9‐point Likert grading scale. Values of 2–8 represented possible intermediate situations. A proposal was deemed appropriate when the value of the median was ≥7 and the score of each rater was ≥5, indicating relative agreement, and strong agreement when the value of the median was ≥7, with no singular rater score <7. In cases of diversity, questions and statements were revised based on comments from the rating group and went to round two. In the first round, the 32 questions were presented to the rating group, and 12 out of 32 questions and statements needed revision after the first round. Therefore, the revised questions and statements had to be rated again by the rating group in the second round. Six of the 12 belonged to Part 1. The results of the two rounds were evaluated by the steering group and, and the statements were modified where needed. The review group was composed of 17 European surgeons selected from European national arthroscopic sports and trauma societies, surgeons who routinely manage patients with patellofemoral instabilities. The review group was specifically required to evaluate the recommendations in the final document for their relevance to a diverse European readership, together with their geographical adaptability and readability. The entire project can also be read in more detail on the ESSKA website (https://esskaeducation.org/esska-consensus-projects).

### Review group

The peer review group, consisting of 17 orthopaedic surgeons appointed by the various arthroscopic and sports surgery societies, was presented with the nearly completed manuscript after it had been revised following the second review by the rating group. The peer review group's comments were incorporated into the final version.

## RESULTS

Out of 32 questions, all 13 questions presented in Part 1 were accepted.

The general median agreement was 8 (range: 7– 9).

There were six with strong agreements and seven with relative agreements.

None were graded A, two were graded B, seven were graded C and four were graded D.

In the review group, there were no objections according to regional rules or laws

(1) Is trauma intensity leading to dislocation or the mechanism of dislocation important for further decision‐making?

There is an inverse correlation between the intensity of trauma leading to FTPD and the underlying pathoanatomic risk factors, meaning that low trauma intensity usually indicates more severe underlying abnormalities. Therefore, the evaluation of trauma intensity provides relevant information in a patient's workup and for clinical decision‐making. Grade: C and rating: median 9 (range 7–9). Strong agreement.

(2a) What are the relevant clinical signs in the acute phase or at a later visit after FTPD?

In the acute phase, examination of the knee might be difficult due to swelling, hemarthrosis and general or localized knee pain. However, the examination should aim to identify whether the patella was dislocated or to detect other types of injury. Patients with suspected hemarthrosis need further magnetic resonance imaging (MRI) investigation as soon as possible and may indicate punction for pain relief. When the acute phase has resolved, a testing protocol consisting of the J‐sign, visual assessment of axial and torsional alignment, range of motion, apprehension test/reversed dynamic patellar apprehension test and patellar glide test is recommended. Grade: D and rating: median 9 (range 5–9). Relative agreement.

(2b) What are the relevant factors in patient history after FTPD?

In addition to the clinical investigation, patient age, family history, bilateral symptoms of instability, and injury mechanism should be evaluated. Grade: B and rating: median 9 (range: 7–9). Strong agreement.

(3) What is the patellofemoral clinical testing protocol after FTPD that is performed in every case?

Examination of the knee after FTPD might be limited in the inflammatory acute phase due to swelling, pain, and patient anxiety. If so, it should be repeated as soon as the acute phase has resolved (from days to weeks) to confirm the initial diagnosis and to assess predisposing factors, including the contralateral knee. The examination should include standing, supine and prone position assessments of coronal and axial deformities; knee joint range of motion; J‐sign; patella gliding; and apprehension/reversed dynamic patella apprehension. This does not exclude the need for systematic examination of other knee structures. Grade: D and rating: median 9 (range 7–9). Strong agreement.

(4) Can we indicate nonsurgical treatment just by clinical examination?

In patients with FTPD, it is strongly recommended not only to rely on clinical examination but also to perform imaging evaluation to diagnose osteochondral fractures, evaluate predisposing factors and skeletal maturity and thereby estimate the risk of persistent instability. Further decision‐making on either surgical or nonsurgical treatment should be based on the assembled information of the patient's medical history, clinical examination and imaging findings. Grade: C and rating: median 9 (range: 5–9). Relative agreement.

(5) Do we need to obtain radiographs and/or MR images for every patient with FTPD? Is the final diagnosis of FTPD a meaningful combination of clinical testing, imaging and patient history?

After FTPD, prompt X‐ray (AP, lateral and axial) and MRI or MRI alone of the knee is considered mandatory to rule out osteochondral fractures and/or bony abnormalities. X‐rays are mandatory in the acute phase only in cases where you do not have access to immediate MRI. However, a clinician might identify an exception in an asymptomatic patient presenting relatively late after the incident with a normal clinical knee examination. The final diagnosis of FTPD and further decision‐making are always meaningful combinations of complete patient workups and should not rely only on images. Grade: C and rating: median 9 (range: 6‐9). Relative agreement.

(6) What is the minimum imaging protocol after FTPD to be performed in every case?

In the acute phase after FTPD, plain radiographs (AP, lateral and axial) and MRI, or MRI alone (if accessible immediately), are the required minimum imaging protocol, to be performed as soon as available. In the chronic phase, precise plain X‐rays (AP, true lateral in 30° flexion and axial) and MRI, or MRI alone, should be performed. However, a clinician might identify an exception in an asymptomatic patient presenting relatively late after the incident with a normal clinical knee examination. Grade: D and rating: median 9 (range: 5–9). Relative agreement.

(7) Which radiologic parameters have to be assessed?

Depending on clinical presentation, the radiological parameters to be measured include patellar height, patellotrochlear overlap, trochlear geometry, and axial alignment (tibial tubercle position and knee rotation). Depending on clinical findings on coronal and rotational alignment, additional imaging evaluations may be necessary. There is currently no consensus on clear cut‐off values for these parameters. Grade: D and rating: median 9 (range: 7–9). Strong agreement.

(8) When do we need more imaging than standard radiographs (AP, lateral and axial) and MRI, which measurements should then be performed?

When clinical examination gives rise to suspicion of valgus deformity, well‐executed, long‐standing x‐rays are recommended. The signs of squinting patella and/or if examination in the prone position shows a difference of more than 30° between internal and external rotation of the hip or clinical suspicion of increased tibial external torsion, further radiological assessment by torsional computed tomography (CT) or MRI investigation is recommended to measure femoral antetorsion, knee rotation and tibial external torsion. Grade: C and rating: median 9 (range: 5‐9). Relative agreement.

(9) When and how should a chondral or osteochondral fracture be diagnosed?

The incidence of chondral or osteochondral fractures is high, especially in paediatric patients. Hemarthrosis/lipohemarthrosis should serve as a warning sign. As chondral lesions in the patellofemoral articular area are important prognostic factors, imaging should start immediately with plain radiographs (ap, lateral, axial) and an MRI or an MRI alone as soon as possible to detect all these chondral and osteochondral lesions and to assess repairability. Grade: C and rating: median 9 (range: 8–9). Strong agreement.

(27) Is non‐operated FTPD a risk factor for ongoing symptoms?

In addition to recurrent instability, a variety of symptoms, such as pain, swelling and giving way, functional and psychological limitations, and a reduction in sports participation, affect 50% of patients, reducing their quality of life. Cartilage lesions start in the first episode, and the severity of the damage correlates with the degree of persistent instability. Grade: C and rating: median 9 (range: 5–9). Relative agreement.

(28) What is the complication rate of surgical treatment in addition to persistent instability?

There are no publications on specific complication rates after surgical treatment of FTPD; however, superposable complication rates of medial patellofemoral ligament (MPFL) reconstructions from cohorts of heterogeneous patients with patellar instability can be expected, and the complication rate is variable. The most common complications are patellofemoral pain, reduced range of motion and patellar fracture. Grade: B and rating: median 9 (range: 8–9). Strong agreement.

(29) Which patient‐reported outcome measures (PROMs) should be used to assess outcomes after FTPD?

The most used PROMs are the Kujala, International Knee Documentation Committee, Knee Injury and Osteoarthritis Outcome Score and Lysholm, which are not specific for patellar instability. The Banff Patellofemoral Instability Instrument 2.0 (BPII) and Norwich Patella Instability (NPI) outcome scores are new scores developed specifically for patients (incl. adolescents) troubled by patellar instability. The BPII 2.0 has been thoroughly tested and found to be valid, reliable and disease‐specific. The consensus committee recommends including the BPII 2.0 and/or NPI scores as a minimum in future studies, knowing that they are not validated for all languages. Grade: C and rating: median 9 (range: 5–9). Relative agreement.

## DISCUSSION

The most important finding or recognition of this consensus is based on the perception that more people with FTPD have persistent problems than has been acknowledged thus far and has been the basis of our paradigm around the evaluation and treatment of these patients. The consequence is that these patients, to a greater extent than before, should undergo thorough assessment and investigation and a detailed clinical examination based on a testing protocol and must, as a minimum, undergo the MRI scan of the knee, both in relation to detecting osteochondral lesions and assessing the underlying predisposing factors. In general, patients who have osteochondral lesions of a size that potentially can be reinserted should undergo surgery. Clinical examinations in acute settings are often difficult, and therefore, a secondary examination is needed when the acute inflammatory phase has resolved. A severely swollen knee after FTPD indicates hemarthrosis due to an eventual osteochondral lesion; therefore, MRI investigation as soon as possible is indicated.

The history leading to patellar dislocation is also considered important since the intensity of the trauma is inversely correlated with the underlying pathoanatomic risk factors. In clinical examinations, the reversed dynamic patellar apprehension test is recognized as a particularly helpful tool for estimating the severity of the underlying pathomorphology. Together, several factors are involved in risk management concerning persistent instability symptoms. This will be more thoroughly reviewed in Part II since it is considered important when planning a treatment strategy.

Despite the lack of evidence for physiotherapy for patients with FTPD, physiotherapy is still recommended in all patients preoperatively when possible. Unless surgery needs to be performed acutely, physiotherapy guide rehabilitation is considered sufficient to achieve a satisfying result and therefore should be prescribed; however, follow‐up by an orthopaedic surgeon after a few months of rehabilitation is recommended. Here, patients' clinical status can be evaluated, with favourable use of more modern PROMs, such as the Banff BPII 2.0 score or the NPI score, since they are recognized as guiding clinicians in quantifying patients' perceptions of patellar instability. A treatment strategy can be planned in consultation with the patient and, eventually, the parents as well.

When planning eventual surgery, there is a consensus that surgery cannot be indicated only based on clinical examination, since every patient requires individual‐based evaluation. FTPD is a symptom that can result from different underlying pathomorphologies, and further radiologic evaluation needs to be performed. The radiological parameters that must be included are complex and are still considered debatable. However, radiologic parameters that evaluate trochlear geometry and the degree of trochlear dysplasia are recognized as important, as are parameters that evaluate the patella and trochlear engagement, such as overlap measures such as the patellotrochlear index, and also the Caton–Deschamps index is considered relevant. Additional investigations, such as CT or MRI of the hip, knee and ankle, focusing on torsional abnormalities are receiving increasing attention and must be addressed when the clinical examination indicates this, and similar methods are applied for coronal axis deviation where long‐leg standing, weight‐bearing radiographs are needed.

Owing to inconsistency and variability in the scientific literature, it was considered important to develop a consensus‐based definition of FTPD to be used in future scientific publications: ‘FTPD is the first‐time event when the patella completely leaves the trochlear groove. The event must be confirmed clinically and/or radiologically’.

Evidence has demonstrated that osteochondral lesions are common and need to be diagnosed early. In most cases, they need to be fixed acutely; however, later fixation can also be indicated. The surgical aspects will be thoroughly reviewed in Part II. It should be stressed that patellofemoral pain is now recognized as a complication for MPFL reconstruction, as most previous articles have failed to register this as a complication [12]. This is because it is not uncommon and is considered of great importance from the patient's perspective [16]. Overall, this consensus has led to several new insights based on fruitful discussions between members of ESSKA's patellofemoral instability group and European orthopaedic surgeons with special expertise and interest in the patellofemoral joint. The findings are expected to contribute to changing the paradigms related to the investigation and treatment of FTPD. Overall, the ESSKA consensus model has been found to be fruitful in the topic of FTPD since the evidence in this area is generally low in grade, although relative agreement (between 5 and 9) and strong agreement (between 7 and 9) were achieved and thereby high level of agreement for most of the statements.

A limitation is the temporal aspect, as the study was conducted over 3 years and since the literature search was completed, new studies may have been published that were not included.

The clinical aspects of this consensus are comprised of a number of different recommendations that contradict previous paradigms. Central to this is the recognition that a much larger proportion of patients with FTPD are observed to have ongoing symptoms despite not experiencing recurrent patellar dislocation, which is related to question no. 27. Another important point related to question no. 28, which has not received much attention, is that a significant proportion of patients are reported to experience persistent patellofemoral pain after MPFL reconstruction despite achieving good stability [1, 2, 3, 6, 7, 8, 9, 10, 13, 15, 17, 18, 19, 20, 23, 24, 25, 26, 27, 28, 29, 30, 31].

It is recommended that the entire consensus paper, including a more thorough review of the literature as well as a more detailed list of references, be read. It can be downloaded from the ESSKA website (https://esskaeducation.org/esska-consensus-projects) and on the ESSKA Academy website (open access).

## CONCLUSION

In relation to the management of patients with FTPD, the ESSKA Patellofemoral Instability Study Group 13 questions have been created and based on these 13 statements was worked on and consensus was reached in cooperation with orthopaedic surgeons representing all Europe geographically.

## AUTHOR CONTRIBUTIONS

All authors (members of the ESSKA Patellofemoral Instability Study Group): Conceptualization and study design, data collection. Manuscript writing and analysis of data: Lars Blønd. Manuscript revision: Peter Balcarek and Philippe Beaufils.

## CONFLICT OF INTEREST STATEMENT

Lars Blønd reports consulting for Arthrex. Peter Balcarek reports consulting for Arthrex. Philippe Beaufils serves as the ESSKA Consensus Projects Advisor. Florian Dirisamer reports consulting for Arthrex and receives royalties from Arthrex Inc. Rene El Attal reports consulting for Arthrex, DepuySynthes and Zimmer Biomet. Geert Pagenstert reports consulting for DepuySynthes and Stryker. Joan Minguell reports consulting for Arthrex and Smith&Nephew. Petri Sillanpaa reports consulting for Inion LTD. Ramazan Akmese reports consulting for Smith&Nephew, and Jacek Walawski reports consulting for Arthrex, Moximed and Smith&Nephew. The remaining authors declare no conflicts of interest.

## ETHICS STATEMENT

This article does not contain any studies with human participants or animals performed by any of the authors.

## Data Availability

The data can be requested from Anna Hansen Rak hansen.anna@esska.org.

## References

[1] Askenberger M , Ekström W , Finnbogason T , Janarv PM . Occult intra‐articular knee injuries in children with hemarthrosis. Am J Sports Med. 2014;42:1600–1606. 24753236 10.1177/0363546514529639

[2] Askenberger M , Janarv P‐M , Finnbogason T , Arendt EA . Morphology and anatomic patellar instability risk factors in first‐time traumatic lateral patellar dislocations. Am J Sports Med. 2017;45:50–58. 27613760 10.1177/0363546516663498

[3] Balcarek P , Jung K , Ammon J , Walde TA , Frosch S , Schüttrumpf JP , et al. Anatomy of lateral patellar instability: trochlear dysplasia and tibial tubercle‐trochlear groove distance is more pronounced in women who dislocate the patella. Am J Sports Med. 2010;38:2320–2327. 20713643 10.1177/0363546510373887

[4] Beaufils P , Dejour D , Filardo G , Monllau JC , Menetrey J , Seil R , et al. ESSKA consensus initiative: why, when and how? J Exp Orthop. 2023;10:1–10. 37801160 10.1186/s40634-023-00664-2 PMC10558408

[5] Beaufils P , Saffarini M , Karlsson J , Hirschmann MT , Prill R , Becker R , et al. High scientific value of consensus is based on appropriate and rigorous methodology: the ESSKA formal consensus methodology. Knee Surg Sport Traumatol Arthrosc. 2025;33:16–20. 10.1002/ksa.12390 39154255

[6] Beckert MW , Albright JC , Zavala J , Chang J , Albright JP . Clinical accuracy of J‐sign measurement compared to magnetic resonance imaging. Iowa Orthop J. 2016;36:94–97. 27528843 PMC4910780

[7] Fabing M , Anderson K . Arthroscopic anterior and posterior capsulolabral advancement for higher‐risk athletes. Oper Tech Sports Med. 2007;15:105–110.

[8] Feller JA , Richmond AK , Wasiak J . Medial patellofemoral ligament reconstruction as an isolated or combined procedure for recurrent patellar instability. Knee Surg Sports Traumatol Arthrosc. 2014;22:2470–2476. 24928369 10.1007/s00167-014-3132-0

[9] Fithian DC , Paxton EW , Stone ML , Silva P , Davis DK , Elias DA , et al. Epidemiology and natural history of acute patellar dislocation. Am J Sports Med. 2004;32:1114–1121. 15262631 10.1177/0363546503260788

[10] Flanigan DC , Shemory S , Lundy N , Stitgen M , Long JM , Magnussen RA . Medial patellofemoral ligament reconstruction with allograft versus autograft tissue results in similar recurrent dislocation risk and patient‐reported outcomes. Knee Surg Sports Traumatol Arthrosc. 2020;28:2099–2104. 32185451 10.1007/s00167-020-05920-x

[11] Frings J , Balcarek P , Tscholl P , Liebensteiner M , Dirisamer F , Koenen P . Conservative versus surgical treatment for primary patellar dislocation. Dtsch Arztebl Int. 2020;117:279–286. 32519945 10.3238/arztebl.2020.0279 PMC7370958

[12] Gorbaty JD , Varkey DT , Hong IS , Trofa DP , Odum SM , Piasecki DP , et al. Patient‐reported outcomes after revision surgery for failed medial patellofemoral ligament reconstruction: a matched‐pair analysis including correction of predisposing factors. Am J Sports Med. 2022;48:3566–3572. 10.1177/0363546520966354 33104394

[13] Hiemstra LA , Kerslake S , Loewen M , Lafave M . Effect of trochlear dysplasia on outcomes after isolated soft tissue stabilization for patellar instability. Am J Sports Med. 2016;44:1515–1523. 27217524 10.1177/0363546516635626

[14] Hysing‐Dahl T , Inderhaug E , Faleide AGH , Magnussen LH . Patients' experiences of living with patellar instability before and after surgery: a qualitative interview study. BMJ Open. 2023;13:e072141. 10.1136/bmjopen-2023-072141 PMC10277117 37295823

[15] Imhoff FB , Funke V , Muench LN , Sauter A , Englmaier M , Woertler K , et al. The complexity of bony malalignment in patellofemoral disorders: femoral and tibial torsion, trochlear dysplasia, TT–TG distance, and frontal mechanical axis correlate with each other (Knee Surgery, Sports Traumatology, Arthroscopy, (2020). Knee Surg Sports Traumatol Arthrosc. 2020;28:897–904. 31127313 10.1007/s00167-019-05542-y

[16] Jackson GR , Tuthill T , Gopinatth V , Mameri ES , Jawanda H , Sugrañes J , et al. Complication rates after medial patellofemoral ligament reconstruction range from 0% to 32% with 0% to 11% recurrent instability: a systematic review. Arthroscopy. 2023;39:1345–1356. 36764559 10.1016/j.arthro.2023.01.098

[17] Kaiser P , Attal R , Kammerer M , Thauerer M , Hamberger L , Mayr R , et al. Significant differences in femoral torsion values depending on the CT measurement technique. Arch Orthop Trauma Surg. 2016;136:1259–1264. 27501703 10.1007/s00402-016-2536-3 PMC4990621

[18] Kaiser P , Schmoelz W , Schöttle PB , Heinrichs C , Zwierzina M , Attal R . Isolated medial patellofemoral ligament reconstruction for patella instability is insufficient for higher degrees of internal femoral torsion. Knee Surg Sports Traumatol Arthrosc. 2019;27:758–765. 30062643 10.1007/s00167-018-5065-5

[19] Lafave MR , Hiemstra L , Kerslake S . Factor Analysis and Item Reduction of the Banff Patella Instability Instrument (BPII): introduction of BPII 2.0. Am J Sports Med. 2016;44:2081–2086. 27166290 10.1177/0363546516644605

[20] Magnussen RA , Verlage M , Stock E , Zurek L , Flanigan DC , Tompkins M , et al. Primary patellar dislocations without surgical stabilization or recurrence: how well are these patients really doing? Knee Surg Sports Traumatol Arthrosc. 2015;25:2352–2356. 26215775 10.1007/s00167-015-3716-3

[21] Martin RK , Leland DP , Krych AJ , Dahm DL . Treatment of first‐time patellar dislocations and evaluation of risk factors for recurrent patellar instability. Sports Med Arthrosc. 2019;27:130–135. 31688530 10.1097/JSA.0000000000000239

[22] McCarthy MI , Hinckel BB , Arendt EA , Chambers CC . Putting it all together: evaluating patellar instability risk factors and revisiting the “menu”. Clin Sports Med. 2022;41:109–121. 34782068 10.1016/j.csm.2021.07.009

[23] Seeley MA , Knesek M , Vanderhave KL . Osteochondral injury after acute patellar dislocation in children and adolescents. J Pediatr Orthop. 2013;33:511–518. 23752148 10.1097/BPO.0b013e318288b7a0

[24] Smith TO , Davies L , O'Driscoll M‐L , Donell ST . An evaluation of the clinical tests and outcome measures used to assess patellar instability. Knee. 2008;15:255–262. 18328714 10.1016/j.knee.2008.02.001

[25] Smith TO , Donell ST , Clark A , Chester R , Cross J , Kader DF , et al. The development, validation and internal consistency of the Norwich Patellar Instability (NPI) score. Knee Surg Sports Traumatol Arthrosc. 2014;22:324–335. 23306714 10.1007/s00167-012-2359-x

[26] Staheli LT . Torsion—treatment indications. Clin Orthop Relat Res. 1989;247:61–66. 2676305

[27] Stuberg W , Temme J , Kaplan P , Clarke A , Fuchs R . Measurement of tibial torsion and thigh‐foot angle using goniometry and computed tomography. Clin Orthop Relat Res. 1991;272:208–212. 1934735

[28] Vollnberg B , Koehlitz T , Jung T , Scheffler S , Hoburg A , Khandker D , et al. Prevalence of cartilage lesions and early osteoarthritis in patients with patellar dislocation. Eur Radiol. 2012;22:2347–2356. 22645041 10.1007/s00330-012-2493-3

[29] White AE , Otlans PT , Horan DP , Calem DB , Emper WD , Freedman KB , et al. Radiologic measurements in the assessment of patellar instability: a systematic review and meta‐analysis. Orthop J Sports Med. 2021;9:1–11. 10.1177/2325967121993179 PMC8142007 34095324

[30] Zhang Z , Zhang H , Song G , Zheng T , Ni Q , Feng H . Increased femoral anteversion is associated with inferior clinical outcomes after MPFL reconstruction and combined tibial tubercle osteotomy for the treatment of recurrent patellar instability. Knee Surg Sports Traumatol Arthrosc. 2020;28:2261–2269. 31797022 10.1007/s00167-019-05818-3

[31] Zimmermann F , Liebensteiner MC , Balcarek P . The reversed dynamic patellar apprehension test mimics anatomical complexity in lateral patellar instability. Knee Surg Sports Traumatol Arthrosc. 2019;27:604–610. 30293182 10.1007/s00167-018-5198-6

